# Topotecan is a potent inhibitor of SUMOylation in glioblastoma multiforme and alters both cellular replication and metabolic programming

**DOI:** 10.1038/s41598-017-07631-9

**Published:** 2017-08-07

**Authors:** Joshua D. Bernstock, Daniel Ye, Florian A. Gessler, Yang-ja Lee, Luca Peruzzotti-Jametti, Peter Baumgarten, Kory R. Johnson, Dragan Maric, Wei Yang, Donat Kögel, Stefano Pluchino, John M. Hallenbeck

**Affiliations:** 1 Stroke Branch, National Institute of Neurological Disorders and Stroke, National Institutes of Health, Bethesda, MD USA; 2 Wellcome Trust-Medical Research Council Stem Cell Institute, Department of Clinical Neurosciences, University of Cambridge, Cambridge, UK; 3 Department of Neurosurgery, Johann Wolfgang Goethe-Universität, Frankfurt am Main, Germany; 4 Edinger Institute, Johann Wolfgang Goethe-Universität, Frankfurt am Main, Germany; 5 Bioinformatics Section, Information Technology & Bioinformatics Program, Division of Intramural Research (DIR), (NINDS/NIH), Bethesda, MD USA; 6 Flow Cytometry Core Facility, National Institute of Neurological Disorders and Stroke, National Institutes of Health (NINDS/NIH), Bethesda, MD USA; 7 Department of Anesthesiology, Duke University Medical Center, Durham, NC USA

## Abstract

Protein SUMOylation is a dynamic post-translational modification shown to be involved in a diverse set of physiologic processes throughout the cell. SUMOylation has also been shown to play a role in the pathobiology of myriad cancers, one of which is glioblastoma multiforme (GBM). As such, the clinical significance and therapeutic utility offered via the selective control of global SUMOylation is readily apparent. There are, however, relatively few known/effective inhibitors of global SUMO-conjugation. Herein we describe the identification of topotecan as a novel inhibitor of global SUMOylation. We also provide evidence that inhibition of SUMOylation by topotecan is associated with reduced levels of CDK6 and HIF-1α, as well as pronounced changes in cell cycle progression and cellular metabolism, thereby highlighting its putative role as an adjuvant therapy in defined GBM patient populations.

## Introduction

Glioblastoma multiforme (GBM) is the most common and aggressive cancer intrinsic to the brain and has therefore been designated by the World Health Organization (WHO) as a grade IV glioma^1, 2^. The diffuse infiltrative growth of GBM tumour cells into adjacent tissue prevents complete tumour resection, resulting in the need for adjuvant therapy^3^ consisting of combined radio- and/or chemotherapy^4–6^. Unfortunately, residual tumour cells display an intrinsic resistance against chemotherapeutic agents^7, 8^. Treatment of these primary brain tumours therefore presents a major challenge for both neurosurgery and clinical neuro-oncology, as the prognosis remains dismal even after maximal surgical resection of the tumour in combination with adjuvant chemoradiation.

Recent advances in the understanding of the signalling pathways that underlie GBM pathogenesis reflect the highly mutated genome of GBM, which is characterized by the dysregulation of many key pathways including those involved in growth, proliferation, survival, metabolism and/or apoptosis^9, 10^. To address the enormous complexity of such dysregulated network dynamics in GBM, a focus on plurifunctional targets that affect multiple pathways is prudent. One such target is global small ubiquitin-related modifier (SUMO)ylation, a post-translational modification (PTM) that operates in states of tolerance and acts to preserve homeostasis under stress^11^. Briefly, SUMO, like ubiquitin, is synthesized as an inactive precursor and is processed by SUMO-specific proteases (SENPs) to yield its mature form^12^. A single heterodimeric E1 enzyme, SAE1/SAE2, serves to initiate conjugation by adenylating SUMO, leading to the formation of a covalent thioester E1-SUMO intermediate. Subsequently, SUMO is then transferred to the catalytic cysteine of the sole E2-conjugase, Ubc9, which either alone or in concert with a target specific E3-ligase catalyses the formation of an isopeptide linkage between the C-terminal glycine residue of SUMO and the ε amino group of the substrate lysine residue. SUMO conjugation is balanced via the deconjugative actions of the various SENPs^13^. There are three systemically distributed SUMO paralogs in mammals: SUMO-2 and SUMO-3, which are 97% identical and cannot be distinguished by specific antibodies; and SUMO-1, which shares only 47% homology with the other paralogs and therefore has distinct immunoreactivity^14^. SUMOylation has been documented to play a role in numerous processes throughout the cell, including signal transduction, gene expression, chromatin remodelling, and protein translocation^14^.

Beyond cellular homeostasis, evidence has emerged to support a critical role for SUMOylation in the development and progression of numerous cancers^15, 16^. Considering the role SUMOylation plays in maintaining cellular function under states of stress/unfavourable conditions, it is not surprising that substantial evidence indicates a positive association between SUMOylation and cancer cell growth, tumorigenesis, metastasis, and, ultimately, poor patient prognosis^17^. Of particular interest are the recent reports that have emerged linking SUMOylation to the development and progression of GBM^18, 19^. Yang *et al*. demonstrated that SUMO-conjugation is activated in human astrocytic brain tumours, with levels of both SUMO-1 and SUMO-2/3 conjugated proteins shown to be markedly elevated in GBM^18^. Blocking SUMO-1-3 conjugation in GBM cells inhibited DNA synthesis, cell growth, and the clonogenic survival of GBM cells^18^. Further, the eloquent work of Bellail and colleagues^19^ definitively demonstrated that cyclin dependent kinase (CDK)6 is modified by SUMO-1 in GBM, and that CDK6 SUMOylation stabilizes the protein and drives the cell cycle, leading to cancer development/progression via inhibition of its ubiquitin-mediated degradation. These findings collectively support the idea that the inhibition of SUMO-conjugation (ideally via an approved/repurposed small molecule) may provide a novel/unique strategy in the treatment of GBM.

The family of camptothecins currently consists of irinotecan, topotecan, and 9-aminocamptothecin^20^, with camptothecin having originally been isolated from the Chinese yew tree, *Camptotheca acuminate* ^21^. Topotecan is a semisynthetic water-soluble derivative of the parent compound camptothecin and is approved by the Food and Drug Administration (FDA) for use in several cancers (e.g. cervical, ovarian, and small cell lung cancer)^22, 23^. The principal mechanism of action for this class of molecules relates to the inhibition of DNA topoisomerase I. Therefore, topotecan exerts the majority of its cytotoxic effects during S-phase of the cell cycle^24^. Given that topotecan has been shown capable of modulating the SUMOylation status of its target DNA topoisomerase I^25^ and has recently been reported to have other non-canonical functions (e.g. the inhibition of hypoxia inducible factor-1 [HIF-1α])^26^, we sought to explore its ability to modulate global SUMOylation in GBM.

Herein we describe topotecan’s ability to act as a novel inhibitor of global SUMOylation and its putative role as an adjuvant therapy in GBM treatment regimens via the inhibition of CDK6 and HIF-1α which promote changes in both GBM cell cycle progression and cellular metabolism.

## Results

### Ginkgolic and anacardic acid fail to decrease levels of protein SUMOylation in human GBM lines

**Figure 1:**
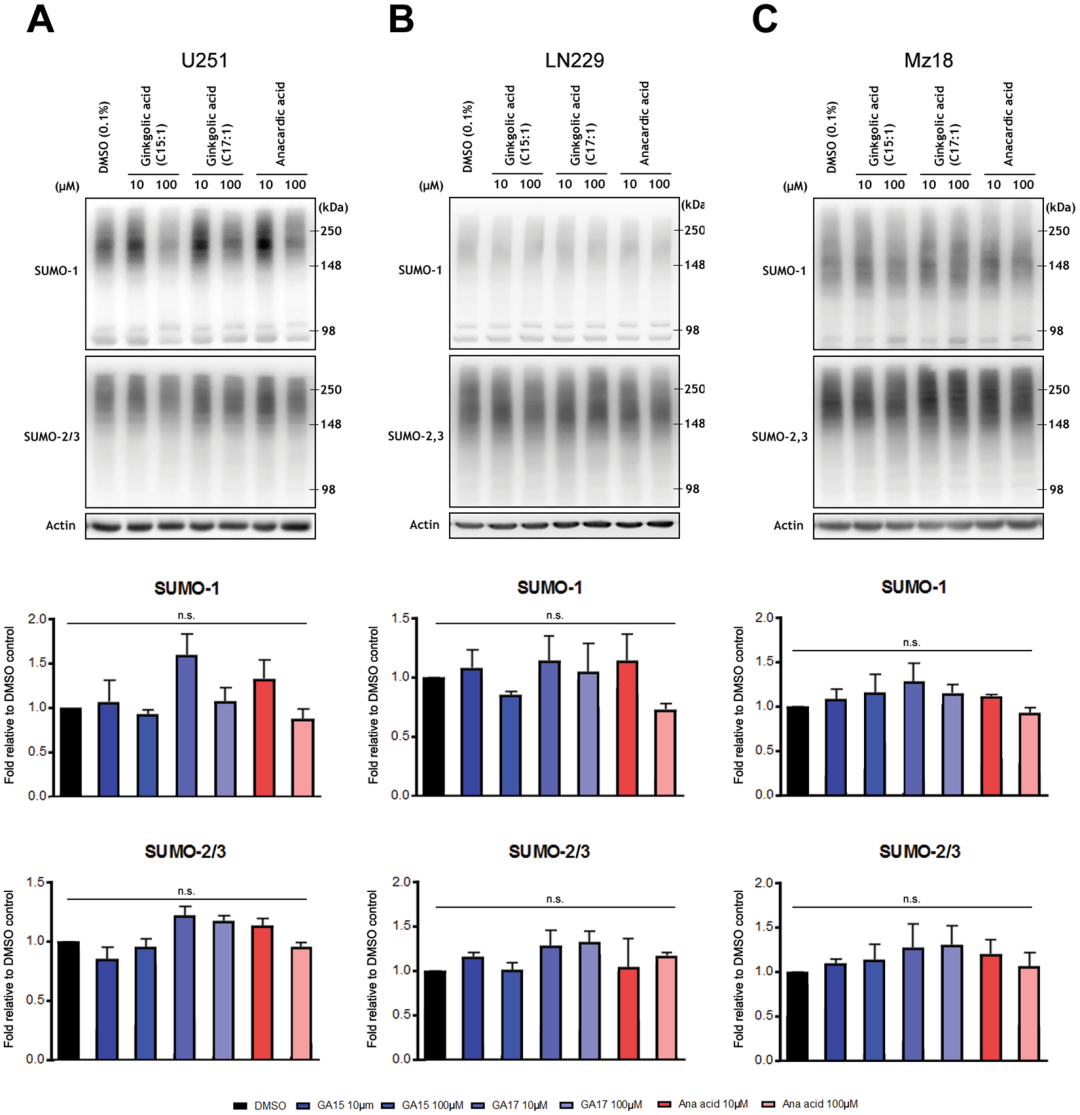
Ginkgolic and anacardic acid fail to suppress global SUMO-conjugation in human GBM lines. U251 (**A**), LN229 (**B**), and Mz18 (**C**), at concentrations of 10 μM or 100 μM. Representative immunoblots are shown. High molecular weight (>100 kDa) SUMO-1 and SUMO-2/3 conjugates were cropped in each lane and the total intensities were measured. Densitometries were normalized to corresponding actin levels and expressed as a fold difference relative to the control (DMSO). Data are means (±SD) from n = 3 independent experiments.

Multiple reports have emerged to link SUMOylation, with the pathogenesis of GBM. Accordingly, the inhibition of SUMOylation via genetic manipulation of the pathway has been shown to be efficacious in preclinical models of GBM^18, 19^. Herein we sought to improve upon such experiments and in so doing extend the translational relevance of such critical work by exploring the ability of reported small molecule inhibitors of SUMOylation in representative human GBM lines (i.e. U251, LN229 and Mz18). Surprisingly, we found that the established SUMOylation inhibitor ginkgolic acid (C15:1)/(C17:1) and its structural analog anacardic acid were not capable of decreasing SUMO-1 or SUMO-2/3-conjugation at either 10 µM or 100 µM concentrations (Fig. 1).

### The alkylating agent temozolomide (TMZ) does not alter SUMO-conjugation in human GBM lines

**Figure 2:**
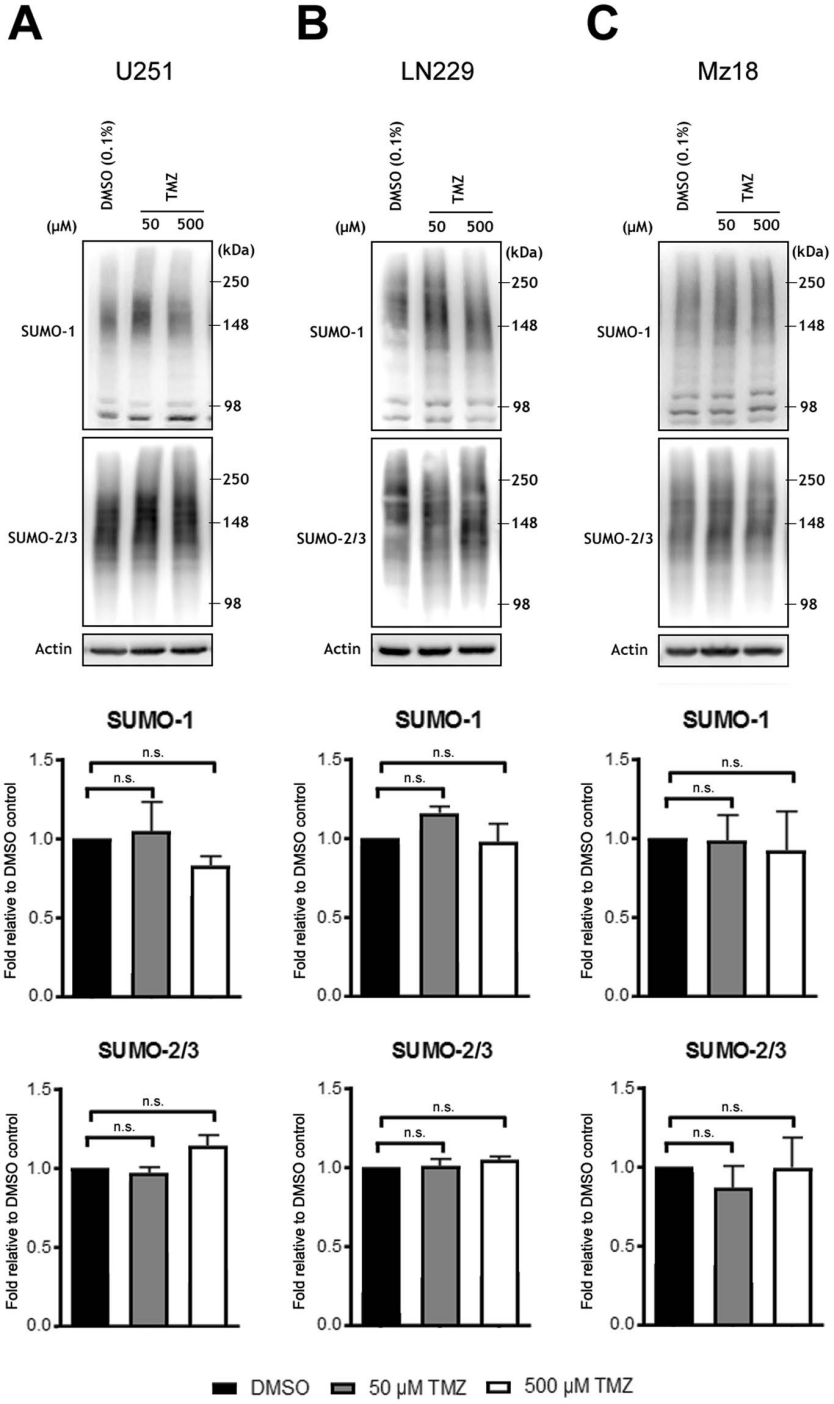
Temozolomide does not decrease global levels of SUMO-conjugation in human GBM lines. U251 (**A**), LN229 (**B**), and Mz18 (**C**), at concentrations of 50 μM and 500 μM. Representative immunoblots are shown. High molecular weight (>100 kDa) SUMO-1 and SUMO-2/3 conjugates were cropped in each lane and the total intensities were measured. Densitometries were normalized to corresponding actin levels and expressed as a fold difference relative to the control (DMSO). Data are means (±SD) from n = 3 independent experiments.

Previous work has indicated that protein SUMOylation is altered in cells exposed to alkylating agents^27^. To further explore global SUMOylation in the context of the standard GBM chemotherapeutic regimen we examined the levels of SUMO-1 and SUMO-2/3 conjugation after exposure to the alkylating agent temozolomide (TMZ) at concentrations of 50 µM and 500 µM; TMZ did not decrease levels of SUMOylation at these doses. In fact, there was a trend towards increased conjugation of SUMO-1 that failed to reach statistical significance compared to the DMSO control (Fig. 2).

### Topotecan inhibits SUMO-conjugation in multiple cancer cell lines and primary rat cortical neurons

**Figure 3:**
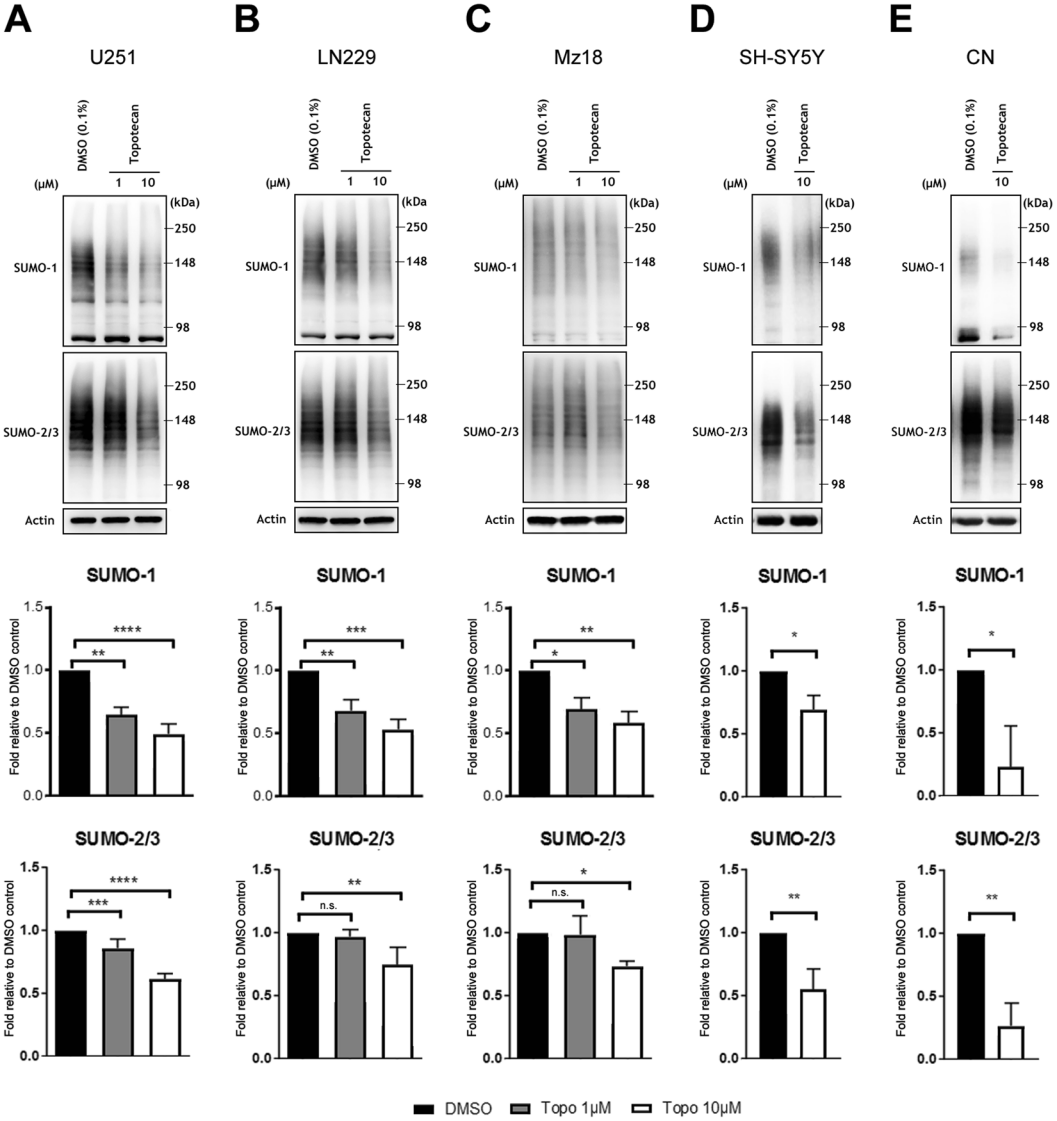
Topotecan causes a decrease in the levels of SUMOylated proteins in GBM, neuroblastoma and E18 primary cortical neurons. U251 (**A**), LN229 (**B**), and Mz18 (**C**) at concentrations of 1 μM and 10 μM, and neuroblastoma line SH-SY5Y (**D**) and rat E18 primary cortical neurons (**E**) at 10 μM. Representative immunoblots are shown. High molecular weight (>100 kDa) SUMO-1 and SUMO-2/3 conjugates were cropped in each lane and the total intensities were measured. Densitometries were normalized to corresponding actin levels and expressed as a fold difference relative to the control (DMSO). Data are means (±SD) from n = 3 independent experiments. *p ≤ 0.05, **p ≤ 0.01, ***p ≤ 0.001, ****p ≤ 0.0001 *vs*. DMSO.

In an effort to effectively target SUMOylation in a manner that would be of translational significance, we utilized the FDA-approved drug topotecan. Topotecan was capable of significantly decreasing the levels of SUMO-1 conjugation at both the 1 µM and 10 µM doses in all three human GBM lines (Fig. 3A,B and C) and SUMO-2/3 conjugation in all three human GBM lines at the 10 µM dose (Fig. 3A,B and C). Of note, topotecan was also capable of inducing a decrease in global SUMO-conjugation in human neuroblastoma SH-SY5Y cells and rat E18-derived primary cortical neurons at 10 µM (Fig. 3D and E). We also examined the effects of topotecan on other PTMs: i.e. Ubiquitin Fold Modifier 1 (UFM1), Neural precursor cell Expressed Developmentally Downregulated-8 (NEDD8), Interferon-Stimulated Gene-15 (ISG15), and Fau Ubiquitin-like protein (FUB1). NEDDylation was decreased by topotecan in lines U251 and LN229 but was unaltered in Mz18 (Supplemental Figs 1A, 2A and 3A). The levels of FUB1, UFM1 and ISG15 conjugation did not change after treatment with topotecan in lines U251 (Supplemental Fig. 1B,C and D), LN229 (Supplemental Fig. 2B,C and D) or Mz18 (Supplemental Fig. 3B,C and D). Of note, the mechanism by which topotecan inhibits SUMOylation is unlikely to involve the levels of the conserved E1 (SAE1/SAE2) or E2 (Ubc9) components of the pathway as these proteins were unaffected after exposure to topotecan (Supplemental Fig. 4).

### Topotecan leads to perturbations in both cell cycle and metabolic profiles of human GBM cell lines

**Figure 4:**
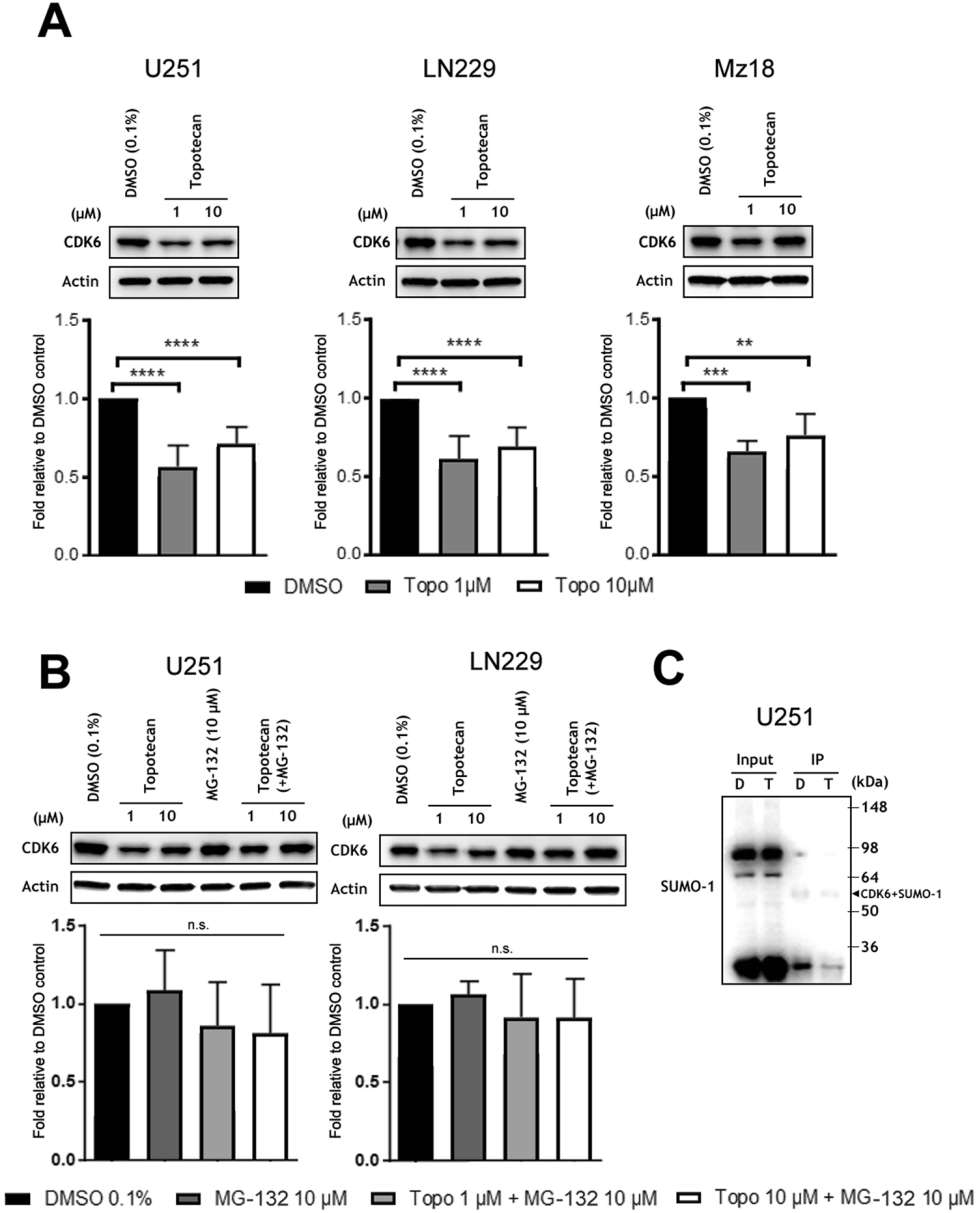
Topotecan alters CDK6 levels in GBM lines. (**A**) Topotecan causes a decrease in CDK6 protein levels in GBM lines U251, LN229, and Mz18 at concentrations of 1 μM and 10 μM. (**B**) The addition of MG132, a specific inhibitor of the protease activity of the 26S complex, attenuates the decrease in CDK6 protein levels previously seen in lines U251 and LN229. Representative immunoblots are shown. The band corresponding to CDK6 (40 kDa) was cropped in each lane and the total intensities were measured. Densities were normalized to corresponding actin levels and expressed as a fold difference relative to the control (DMSO). Data are means (±SD) from n = 5 independent experiments. **p ≤ 0.01, ***p ≤ 0.001, ****p ≤ 0.0001 *vs*. DMSO. (**C**) Lysates from U251 cells treated either with DMSO or topotecan at 1 μM for 12hr were immunoprecipitated using an anti-CDK6 antibody followed by immunoblotting using an anti-SUMO-1 antibody. Arrow highlights the predicted CDK6-SUMO-1 band.

**Figure 5:**
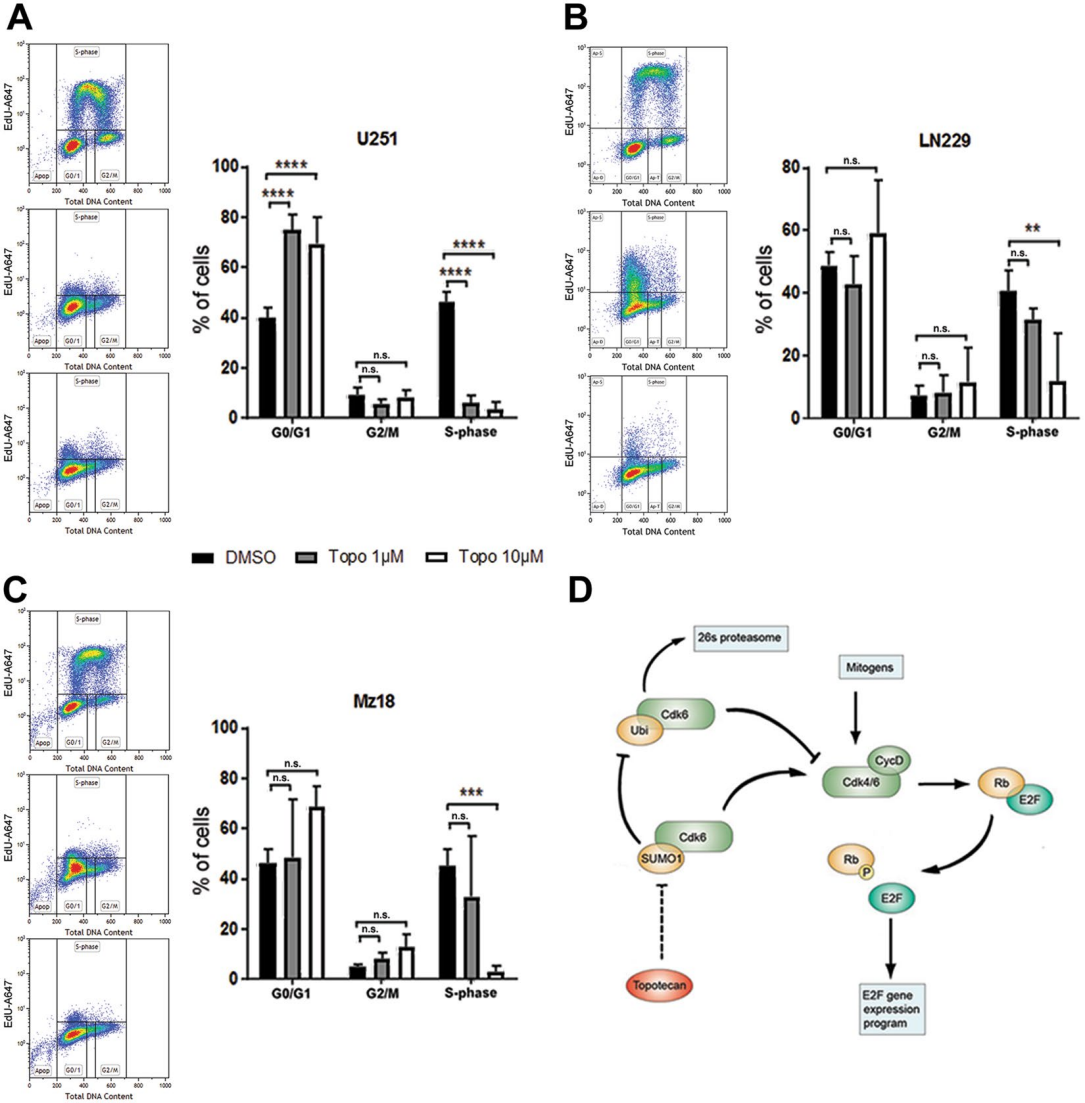
Topotecan alters the cell cycle in GBM lines effecting a decrease in S-phase progression in U251 (**A**), LN229 (**B**), and Mz18 (**C**) and an increase in G0/G1-phase restriction in U251 (**A**). FACS was used to determine the phase of the cell cycle via the incorporation of 5-ethynyl-2′-deoxyuridine (EdU). Data are means (±SD) from n = 3 independent experiments. **p ≤ 0.01, ***p ≤ 0.001, ****p ≤ 0.0001 *vs*. DMSO. (**D**) Putative mechanism by which topotecan perturbs the CDK6 axis and cell cycle progression at the 1 μM and 10 μM doses.

**Figure 6:**
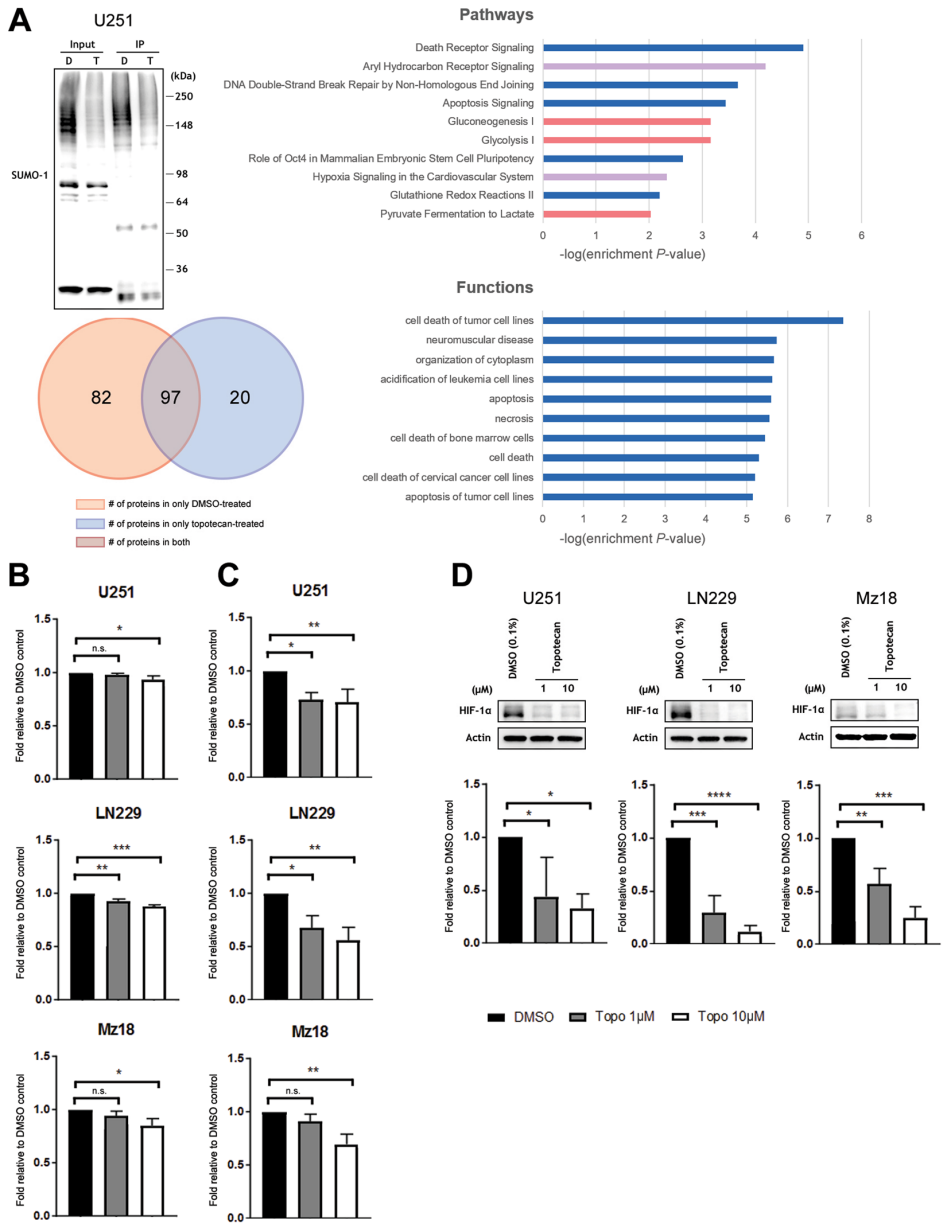
Topotecan alters the proteomic/metabolic profile of GBM cell lines and decreases the levels of HIF-1α. (**A**) Global SUMO-1 pull-down in line U251, probed with an anti-SUMO-1 antibody. D = DMSO (0.1%), T = Topotecan 1 μM. Input = precleared lysate, IP = immunoprecipitated lysate. Mass spectrometry detected 82 proteins co-immunoprecipitated with SUMO-1 in only the DMSO control condition, 20 proteins in only the topotecan condition, and 97 proteins in both conditions. The top 10 attenuated pathways and functions in topotecan-treated cells based on mass spectrometry results are reported; Fisher’s Exact Test was used to assess significance; *x*-axes represent the −log(enrichment P-value). (**B**) Levels of lactate, a by-product of glycolysis, decreases in a dose-dependent manner with topotecan treatment in lines U251, LN229, and Mz18. (**C**) Activity of glucose-6-phosphate dehydrogenase, an enzyme in the pentose phosphate pathway, decreases in a dose-dependent manner with topotecan treatment in lines U251, LN229, and Mz18. (**D**) Topotecan effects a dose-dependent decrease on levels of HIF-1α protein in GBM lines U251, LN229, and Mz18 at concentrations of 1 μM and 10 μM. Representative immunoblots are shown. The bands corresponding to HIF-1α (~116 kDa) were cropped in each lane and the total intensities were measured. Densities were normalized to corresponding actin levels and expressed as a fold difference relative to the control (DMSO). Data in B-D are means (±SD) from n = 3 independent experiments. *p ≤ 0.05, **p ≤ 0.01, ***p ≤ 0.001, ****p ≤ 0.0001 *vs*. DMSO.

We also observed that topotecan reduced the levels of the cell cycle protein CDK6 (Fig. 4A). This decrease is in line with reports by Bellail *et al*.^19^, which demonstrated that CDK6 stability/protection from the proteasome in GBM was dependent on SUMOylation. The addition of the 26S proteasome inhibitor MG132 blocked topotecan mediated CDK6 degradation (Fig. 4B). Furthermore, we conducted immunoprecipitation (IP) experiments in which CDK6 was pulled-down and probed with a polyclonal anti-SUMO1 antibody. Significantly less SUMO-1 was conjugated to CDK6 in GBM cells treated with topotecan (Fig. 4C). Cell cycle analysis revealed a G1/S transition block in GBM cells treated with topotecan in the absence of a major sub-G1 cell fraction indicative of apoptosis. However, topotecan proved to be additive in terms of cellular cytotoxicity in combination with TMZ at 72hrs in the TMZ-resistant line LN229^28^ (Supplemental Fig. 5). Of note, at both the concentrations/times used throughout this study, topotecan does not appear to act solely via inhibition of topoisomerase I^23^, as no significant arrest at the G2/M checkpoint of the cell cycle was observed during fluorescence-activated cell sorting (FACS) analysis (Fig. 5A,B and C). Figure 5D highlights the proposed mechanism of action of topotecan with regard to the CDK6 axis. In an effort to delineate the identity of other proteins which are SUMOylated in GBM we performed global SUMO-1 IPs and subjected those proteins that were pulled-down to liquid chromatography (LC)/mass spectrometry (MS)/MS analysis. The target identification results obtained using line U251 suggested that perturbation of SUMOylation may affect cellular metabolism, which is supported by the literature (Fig. 6A)^29, 30^. Appropriately, both the pentose phosphate pathway and glycolytic metabolism were altered following treatment with topotecan, as suggested by a reduction in lactate levels (Fig. 6B) and glucose-6-phosphate dehydrogenase (G6PD) activity (Fig. 6C). Critically, these results are likely to be related to the dose-dependent decreases identified in HIF-1α after treatment with topotecan in all three GBM lines examined (Fig. 6D).

### Immunohistochemistry (IHC) of surgically resected tumour samples reveals differential levels of SUMOylation between GBM patients

**Figure 7:**
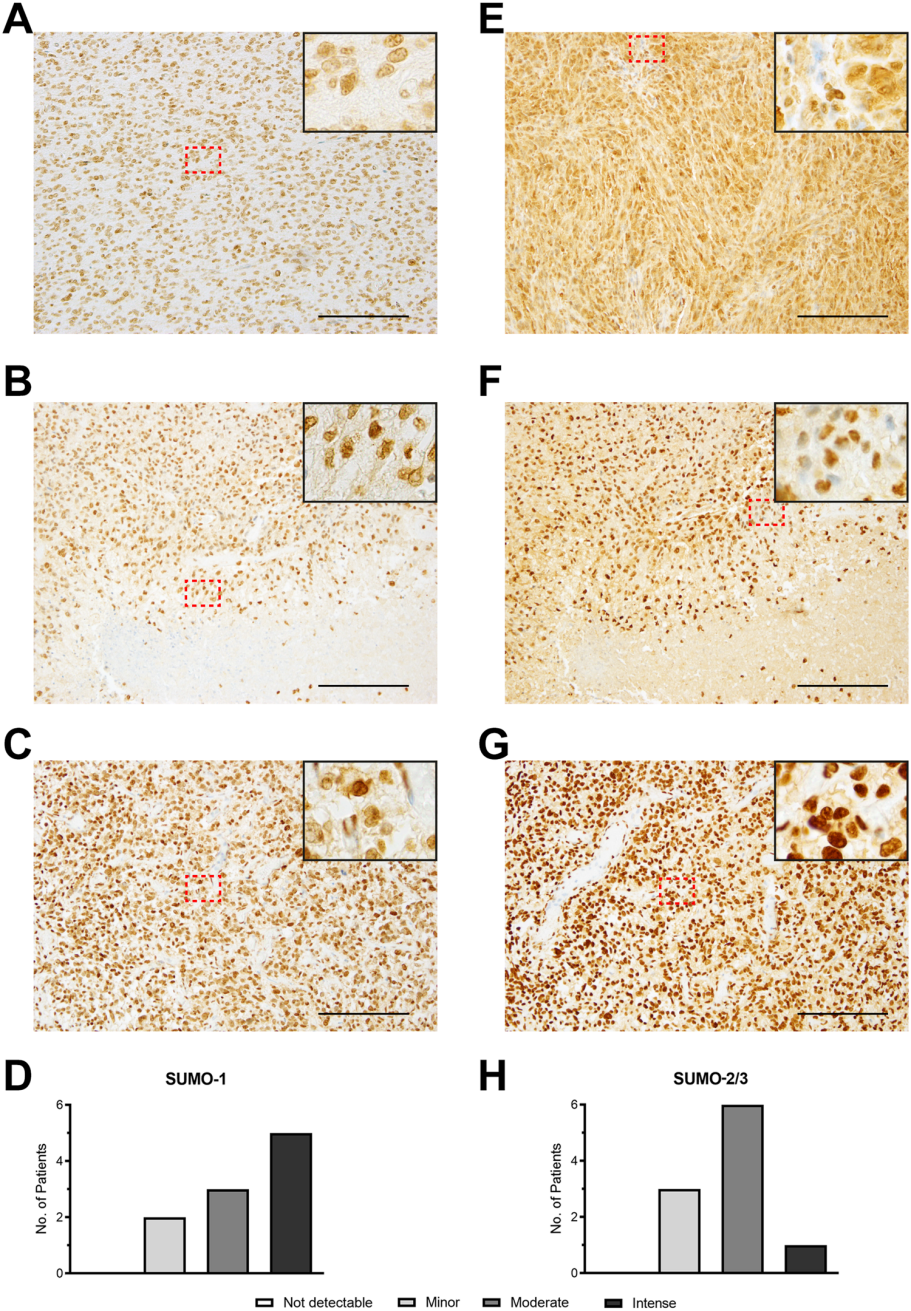
Immunohistochemistry (IHC) reveals different patterns of global SUMOylation between GBM patients. Immunohistochemistry for SUMO-1 and SUMO-2/3 was visualized using 3,3′-diaminobenzidine (DAB) and counterstained with hematoxylin (blue). SUMO-1 expression in GBM showing minor (**A**), moderate (**B**) or intense (**C**) expression. (**D**) Neuropathological scoring of SUMO-1 expression in 10 GBM patients. SUMO-2/3 expression in GBM showing minor (**E**), moderate (**F**) or intense (**G**) expression. (**H**) Neuropathological scoring of SUMO-2/3 expression in 10 GBM patients. Scale bars indicate 200 µm. Images were taken at 10x magnification with enlarged panels representing 40x magnification.

Having shown that topotecan is able to target SUMOylation in a manner that would be of translational importance we sought to extend our findings by confirming the presence of elevated levels of global SUMOylation within certain subpopulations of GBM patients. Accordingly, immunohistochemistry (IHC) for SUMO-1 was performed using samples from 10 GBM patients. Figure 7A represents staining deemed to be minor (2 patients); Fig. 7B represents staining deemed to be moderate (3 patients); Fig. 7C represents staining deemed to be intense (5 patients). Figure 7D confirms that SUMO-1 was detectable in all samples analysed. IHC for SUMO-2/3 expression in GBM was also performed in the same 10 GBM patients. Figure 7E represents staining deemed to be minor (3 patients); Fig. 7F represents staining deemed to be moderate (6 patients); Fig. 7G represents staining deemed to be intense (1 patients). Figure 7H confirms that SUMO-2/3 was detectable in all samples analysed.

## Discussion

Therapeutic options for the treatment of GBM are limited, and despite years of focused research GBM patient prognosis remains dismal. Accordingly, herein we describe the ability of the FDA approved drug topotecan to act as a novel inhibitor of global SUMOylation, as well as its putative role as an adjuvant therapy in GBM treatment regimens via the inhibition of global SUMOylation, CDK6, and HIF-1α.

Critically, dysregulation of SUMOylation has been reported to be involved in tumorigenesis^19, 31–33^; the role of SUMOylation in the majority of cancers appears to be related to an upregulation of the pathway, although downregulations have been described^15, 33^. Therefore, the therapeutic targeting of protein SUMOylation may represent a novel approach for the treatment of myriad cancers, including GBM; interestingly recent reports have also emerged demonstrating that elevations in the conjugation of NEDD8 are involved in the pathogenesis of GBM^34^. In human astrocytic brain tumours, SUMO1–3 conjugation has been shown to be elevated and is required for glioblastoma cell survival^18^; it also serves to modify/stabilize CDK6^19^. Notwithstanding the importance of SUMOylation in regulating diverse biological systems and diseases, only a few small molecule inhibitors of SUMOylation have been reported to date, notably ginkgolic acid and its analog anacardic acid^35^. As such, we analysed these previously reported inhibitors of protein SUMOylation in three different human GBM cell lines. While others have reported decreases in protein SUMOylation using ginkgolic/anacardic acid in various models of cancer (e.g. breast, colorectal and pancreatic), the literature lacks reports on the preclinical efficacy of ginkgolic/anacardic acid in GBM^36, 37^. Incubation of GBM tumour cell lines with ginkgolic acid or anacardic acid did not result in statistically significant decreases in global protein SUMOylation; whether this may be attributed to exceptionally high levels of global SUMOylation in GBM cells of up to ~40-fold of that of normal tissue^18^, the employment of different dosing stratagems and/or some other unique feature of GBM pathobiology remains to be elucidated.

While the camptothecin derivative topotecan continues to be evaluated as a treatment for GBM in ongoing clinical studies (e.g. ClinicalTrials.gov Identifiers: NCT01931098, NCT01004874 NCT00638898), its ability to inhibit SUMOylation has yet to be explored. Here we have demonstrated that topotecan decreases the levels of global SUMOylation in both human GBM and neuroblastoma cell lines. We have also shown that topotecan is capable of inducing a decrease in the levels of global SUMOylation in E18 rat primary cortical neurons, suggesting that the observed changes are not to be solely attributed to the inhibition of topoisomerase I, as differentiated cortical neurons are quiescent (i.e. remaining within the G0 phase of the cell cycle). Critically the reductions in SUMOylation noted throughout the course of this study do not appear to involve the levels of E1/E2 enzymes (SAE1/SAE2 and Ubc9 respectively). Additional work will therefore be required to examine the activity of Ubc9, E3-target specific ligases and/or relevant SENPs after exposure to topotecan in an effort to further elucidate the governing mechanisms of action.

In line with such findings we also observed a concordant decrease in CDK6 levels and alterations in the cell cycle of all three GBM lines examined confirming previous work which has highlighted the role of SUMO1–conjugation in CDK6 stability/cell cycle progression in GBM^19^. By inducing CDK6 degradation via the inhibition of SUMO1-conjugation, topotecan may ultimately enhance the therapeutic efficacy of CDK4/6 kinase inhibitors such as PD0332991 (i.e. palbociclib), which have already displayed promising results in GBM^38^. Such a finding would echo the conclusions put forth by Hamilton *et al*.^39^ in drug-resistant small cell lung cancer, in which they highlight the synergism of CDK inhibitors with camptothecin derivatives, albeit without a definitive mechanism.

It is perhaps unsurprising that novel mechanisms for topotecan exist as other recent reports have shown that topotecan is capable of inhibiting the transcription factor HIF-1α^26, 40^ which is a key driver of GBM tumour progression/therapeutic resistance^41^. Of note, HIF-1α can be SUMOylated and hypoxia is capable of influencing this process^42–47^. It should be noted that controversy exists regarding the underlying cellular mechanisms/outcomes of HIF-1α SUMOylation. Some reports have claimed that SUMOylation stabilizes HIF-1α and in so doing enhances its transcriptional activity, while other reports have claimed that hypoxia-induced HIF-1α SUMOylation negatively regulates its stability and transactivation^43, 45, 46, 48–51^. Here we have shown that topotecan is capable of decreasing HIF-1α levels in all GBM cell lines examined. HIF-1α induces the expression of genes encoding proteins that enable cell survival in hypoxic conditions via an induction of glycolytic enzymes and growth factors (e.g. vascular endothelial growth factor [VEGF] and erythropoietin) that increase vascular supply to the tumour and is therefore a critical oncotarget^40^. Further, HIF-1α has been linked to the expression of epithelial-mesenchymal transition (EMT)-associated proteins in GBM^52^ with EMT itself having shown to be driven primarily by a transcriptional profile controlled via SUMOylation^16^. With regard to cellular metabolism it is important to note that GBM displays a unique bioenergetic state of aerobic glycolysis known as the “Warburg effect”^53, 54^. Accordingly, reports have emerged which have come to suggest that targeting the Warburg shift may represent a novel therapeutic approach for the treatment of GBM^55–57^. Again, both SUMOylation and HIF-1α have been shown to play definite roles in a switch to glycolysis in states of both health and disease^29, 30, 58, 59^. Topotecan’s ability to target both of these axes speaks to its potential as an adjuvant therapy within hypoxic tumour microenvironments and immediately suggests potential synergy with anti-angiogenetic and/or metabolic therapies.

It is prudent to note that topotecan and other camptothecin derivatives have undergone clinical trials in GBM patient cohorts and thus far have displayed limited efficacy^60–65^. As per the differential staining demonstrated upon IHC varying levels of both SUMO-1 and SUMO-2/3 expression exist within GBM patients. To this end, it remains to be determined whether those patients demonstrating a response to topotecan treatment do in fact have elevated levels of SUMOylation. Beyond topotecan, combinations of irinotecan and temozolomide have been examined and appear to display some beneficial effects^66, 67^. Interestingly, a recently published trial which employed a combination therapy centred on bevacuzimab (i.e. a humanized anti-VEGF monoclonal antibody) and irinotecan resulted in a superior progression free survival (PFS) rate and median PFS as compared with temozolomide^68^. Such a finding speaks to the potential synergy of camptothecin derivatives in combination with antiangiogenic therapies with the understanding that both SUMOylation and HIF-1α directly contribute to hypoxic stress responses.

## Conclusions

Topotecan decreases the levels of global SUMO-conjugation, CDK6, and HIF-1α in GBM cells thereby altering both the cell cycle and metabolic profile. Our findings therefore suggest a novel mechanism of action for topotecan and a therapeutic role for the drug in GBM and other cancers, which have hijacked the SUMOylation process (with the understanding that topotecan has FDA approval for the treatment of ovarian, cervical, and small cell lung cancers). It is therefore the authors’ contention that the repositioning of topotecan as an adjuvant therapy in GBM may ultimately lead to improved outcomes in defined cohorts of patients.

## Materials and Methods

### Small molecules

All small molecules (topotecan, anacardic acid, ginkgolic acid, temozolomide, MG132) were purchased from Sigma-Aldrich (Sigma-Aldrich, St. Louis, MO) and stock solutions were made in dimethyl sulfoxide (DMSO). All dilutions resulted in a final concentration of 0.1% DMSO within the growth media.

### Cell culture isolation of primary cortical neurons

The human GBM cell lines (U251, LN229, Mz18) and human neuroblastoma cell line (SH-SY5Y) were cultured in Dulbecco’s Modified Eagle’s Medium (DMEM) (Life Technologies, Grand Island, NY) supplemented with 10% heat-inactivated foetal bovine serum (FBS) (Atlanta Biologicals Inc., Flowery Branch, GA), 100 U/mL penicillin, and 100 mg/mL streptomycin in 5% CO_2_ at 37 °C. Animal experiments were approved by the NIH/NINDS ACUC (Animal Care and Use Committee): cortical neurons were isolated from E18 embryos of Sprague-Dawley rats in accordance with the policies set forth by the ACUC of the NINDS (ASP #1225–14). Further, all experiments utilizing these cells were performed in accordance with NINDS/NIH and ACUC guidelines. Briefly, cortices were dissected from the embryos, dissociated with papain (Worthington Biochemicals, Lakewood, NJ), and plated out at 1,000,000 cells per well on poly-L-lysine-coated 6 well plates in Neurobasal-A/B27 (Life Technologies) media as described previously^69^. Cortical neurons were used after 7–10 days in culture.

### GBM patient tissue microarray (TMA)

Formalin-fixed, paraffin-embedded tissue samples of 10 GBM patients originally diagnosed between 2007–2009 were retrieved from the archives of the Edinger Neurological Institute as part of a Tissue MicroArray (TMA)^70^. Of note, all samples were reviewed by at least two board certified neuropathologists according to the WHO criteria for CNS tumours.

### Cell cycle analysis

350,000 GBM cells/well were plated into 6-well plates and allowed to attach before being treated with topotecan in 0.1% DMSO for 12 hrs. Cells were lifted, fixed, permeabilized, and stained using the Click-iT EdU Plus Flow Cytometry Assay Kit (Thermo Fisher Scientific, Waltham, MA) as per the manufacturer’s instructions. This was followed by counterstaining with 4′,6-diamidino-2-phenylindole (DAPI) to assay total DNA content per cell. Cells were then analysed by flow cytometry using a MoFlo Astrios cell sorter with Summit Acquisition software (Beckman Coulter, Indianapolis IN); 50,000 events were acquired per sample. Data analysis was completed with Kaluza software (Beckman Coulter).

### Immunoprecipitation and Western blot analyses

After removal of the treated media and washing with phosphate buffered saline (PBS), cells were lysed in an IP buffer which contained (50 mM HEPES pH 7.3, 100 mM NaCl, 1.5 mM MgCl2, 1% NP40, 0.1% SDS, 1 mM PMSF, 20 mM NEM, MS-SAFE protease/phosphatase inhibitor cocktail). The cell lysates were then incubated for 1 hr on ice and then centrifuged for 20 min at ~12,000 × g at 4 °C. After pre-clearing with Dynabeads Protein G (Thermo Fisher Scientific), protein concentrations were measured via a Pierce BCA Protein Assay (Thermo Fisher Scientific). Equal amounts of protein amongst the relevant samples were ultimately used for IP. After incubation with the primary antibody for 2 hr at 4 °C, Dynabeads Protein G was added and incubated overnight at 4 °C. The washing and eluting of IP products that followed conformed entirely to the manufacturer’s (Thermo Fisher Scientific) protocol. Total cell lysates for Western blots from SHSY5Y, B35, or E18 primary cortical neurons were prepared as has been previously described^11, 47^. The antibodies used throughout the course of this study were as follows: rabbit polyclonal anti-SUMO-1 and anti-SUMO-2/3 (both developed in-house); anti-SAE1, anti-SAE2, anti-Ubc9, anti-UFM1, and anti-ISG15 (Abcam, Cambridge, MA); anti-NEDD8 (Cell Signaling Technology, Danvers, MA); anti-FUB1 (Abnova, Taipei, Taiwan); anti-CDK6 and anti-HIF-1α (Santa Cruz Biotechnology, Dallas, TX); and anti-β-actin (Sigma-Aldrich). Protein expression levels were determined via densitometric analysis of the corresponding protein bands of interest using ImageJ (NIH, Bethesda, MD). In order to measure SUMO-conjugation levels, regions corresponding to molecular weights above 100 kDa in each lane were cropped and the total intensity analysed. Other PTMs were analysed as previously described^71^. All densities were normalized to the corresponding actin levels and expressed as the ratio to control (DMSO alone).

### Immunohistochemistry

Tumour sections (3 µm) were subjected to IHC for SUMO-1 and SUMO-2/3 (antibodies: in-house rabbit polyclonals, dilutions 1:50). Tissue labelling for all antigens was performed using DiscoveryXT IHC system (Ventana, Strasbourg, France) via standardized protocols as has been previously published^70^. Imaging was performed using an Olympus BX50 light microscope (Olympus, Hamburg, Germany). Intensity was quantified independently by two authors as either “not detectable; minor; moderate; intense”.

### Proteomic identification (LC/MS/MS) and biological function and pathway enrichment analysis

Protein identification of excised Coomassie Blue-stained gel bands was performed by in-gel tryptic digestion, analysis of the resulting digest by LC/MS/MS, and database searching as described previously^72^. MS-identified proteins for two separate SUMO-1 pull-down fractions representing DMSO treatment and TOPO treatment were filtered to keep proteins having at least 2 significant peptide matches. For these proteins, three lists were organized by symbol: detected in both the DMSO and TOPO fractions (n = 97), detected in the DMSO fraction only (n = 82), and detected in the TOPO fraction only (n = 20). For proteins detected in the DMSO fraction only, symbols were imported into Ingenuity Pathway Analysis (IPA) (www.ingenuity.com) and the corresponding enriched functions and pathways identified.

### Lactate levels assays

Cell media were harvested/centrifuged at ~1750 × g after incubation with topotecan for 12 hrs. Samples were subsequently incubated at 99 °C for 10 min to remove the confounding effects of lactate dehydrogenase present within the media/FBS. The lactate fluorometric assay kit was carried out according to the manufacturer’s instructions (BioVision, Milpitas, CA).

### Glucose-6-phosphate dehydrogenase assay

Cells in 10-cm tissue culture plates were treated for 12 hrs with DMSO or topotecan (1 and 10 μM at 0.1% DMSO total volume). After removal of the treated media and washing with PBS, cells were lysed in IP lysis buffer (as per the above) and kept on ice for 1 hr with intermittent inversion. Lysates were then centrifuged at ~12000 × g for 20 min at 4 °C and the pellet was discarded. BCA protein quantitation was used to normalize the amount of protein between samples. The glucose-6-phosphate dehydrogenase activity colorimetric assay kit was carried out according to the manufacturer’s instructions (BioVision).

### Lactate dehydrogenase cytotoxicity assay

Cells were seeded at a density of 200,000 cells/well into 6-well plates and allowed to grow overnight (O/N). Cells were then incubated in media containing increasing doses of TMZ, topotecan, or both at a final concentration of 0.1% DMSO. Readouts were performed at 12 and 72 hrs as per the manufacturer’s directions (LDH Cytotoxicity Kit, Abcam).

### Statistical analysis

To test for differences in treatments (e.g. topotecan) vs control (DMSO), ANOVA (one or two-way) followed by post-hoc testing (Dunnett’s or Bonferroni’s respectively), was performed. Values of p ≤ 0.05 were deemed to be significant.

## Electronic supplementary material

Supplementary Information
