# Supplementary material for: Topotecan is a potent inhibitor of SUMOylation in glioblastoma multiforme and alters both cellular replication and metabolic programming
Source: Sci Rep. 2017 Aug 7;7:7425. doi: 10.1038/s41598-017-07631-9 (PMC5547153; doi:10.1038/s41598-017-07631-9)
Supplement: Supplementary file 1 — Supplementary Information [file 41598_2017_7631_MOESM1_ESM.pdf]

Topotecan is a potent inhibitor of SUMOylation in glioblastoma multiforme and alters both cellular replication and metabolic programming

Joshua D. Bernstock[1,2*], Daniel Ye[1*], Florian A. Gessler[2,3*], Yang-ja Lee[1], Luca Peruzzotti-Jametti[2], Peter Baumgarten[4], Kory R. Johnson[5], Dragan Maric[6], Wei Yang[7], Donat Kögel[3‡], Stefano Pluchino[2‡], John M. Hallenbeck[1‡]

[1]Stroke Branch, National Institute of Neurological Disorders and Stroke, National Institutes of Health, Bethesda, MD, USA

[2]Wellcome Trust-Medical Research Council Stem Cell Institute, Department of Clinical Neurosciences, University of Cambridge, Cambridge, UK

[3]Department of Neurosurgery, Johann Wolfgang Goethe-Universität, Frankfurt am Main, Germany

[4]Edinger Institute, Johann Wolfgang Goethe-Universität, Frankfurt am Main, Germany

[5]Bioinformatics Section, Information Technology & Bioinformatics Program, Division of Intramural Research (DIR), (NINDS/NIH), Bethesda, MD, USA

[6]Flow Cytometry Core Facility, National Institute of Neurological Disorders and Stroke, National Institutes of Health (NINDS/NIH), Bethesda, MD, USA

[7]Department of Anesthesiology, Duke University Medical Center, Durham, NC, USA

[*]These authors contributed equally to this work

[‡]These authors contributed equally to this work

Supplemental Figure 1

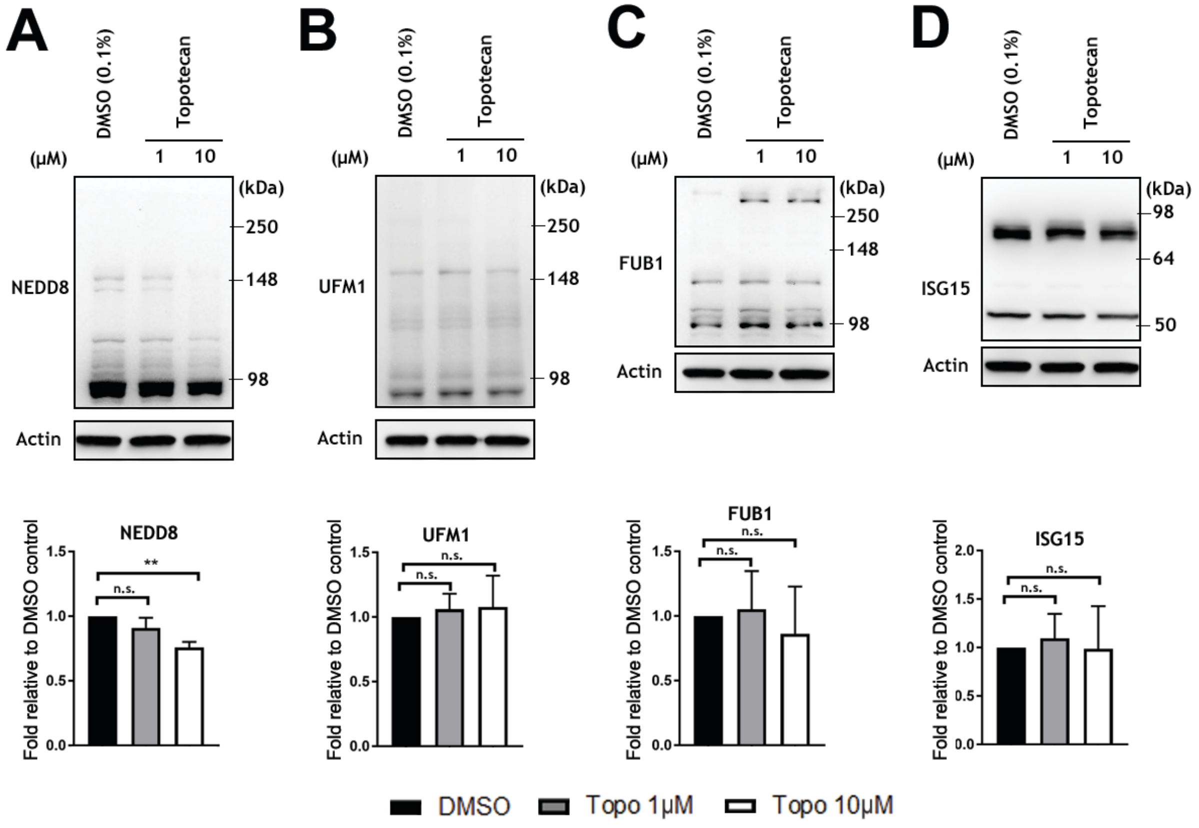

Supplemental Figure 2

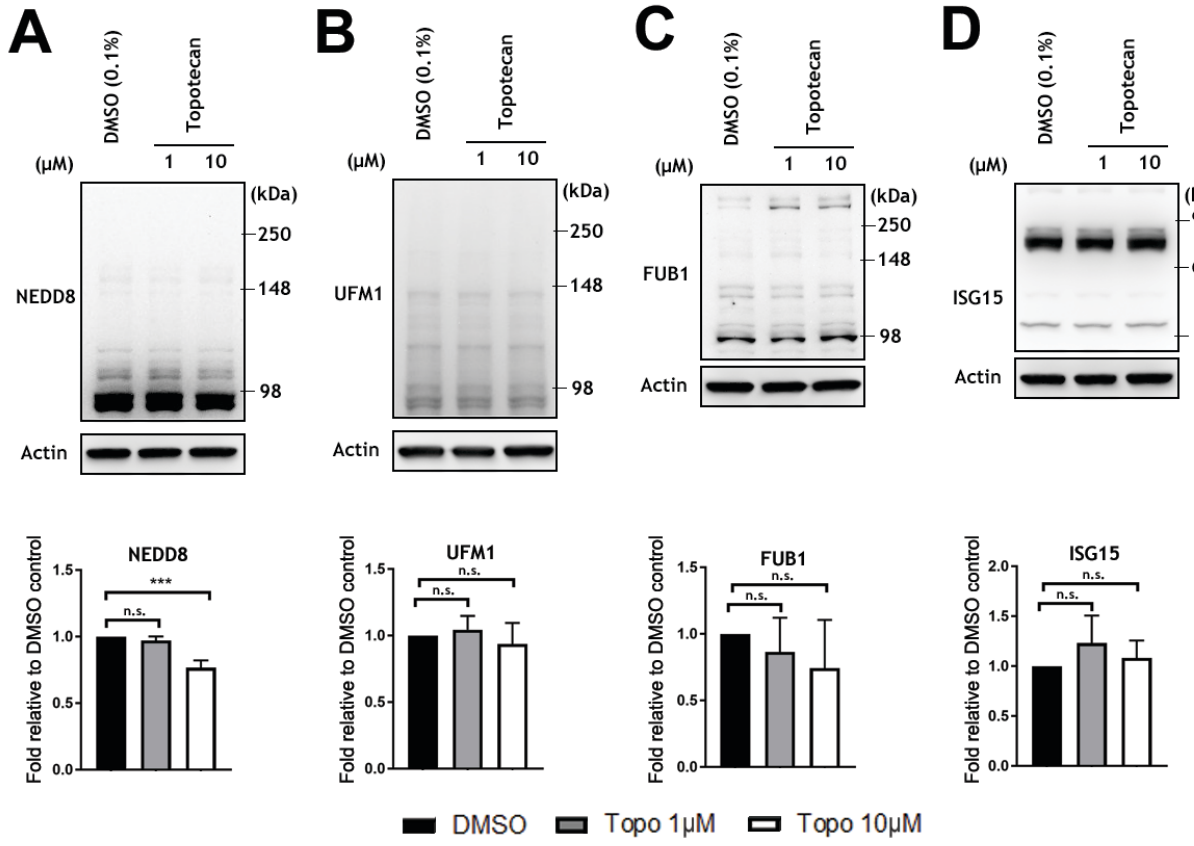

Supplemental Figure 3

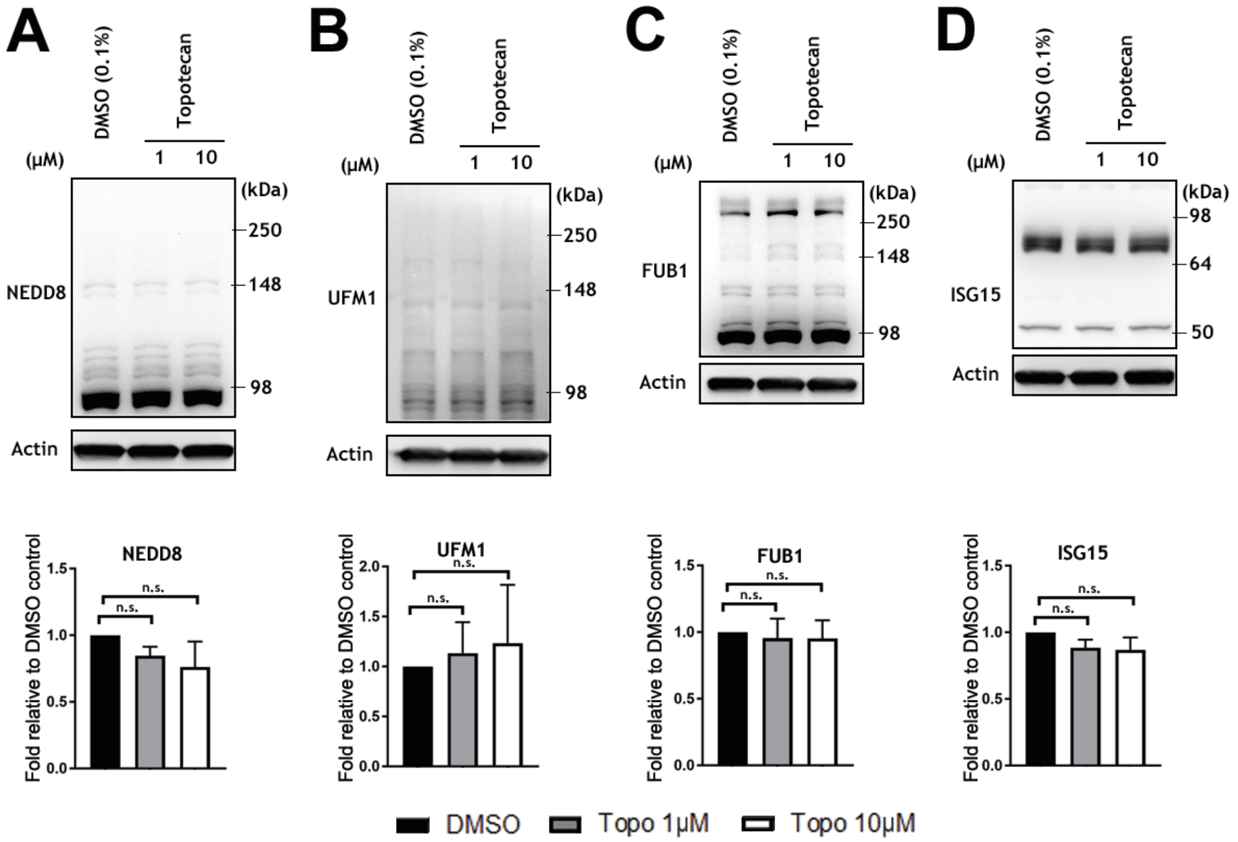

Supplemental Figure 4

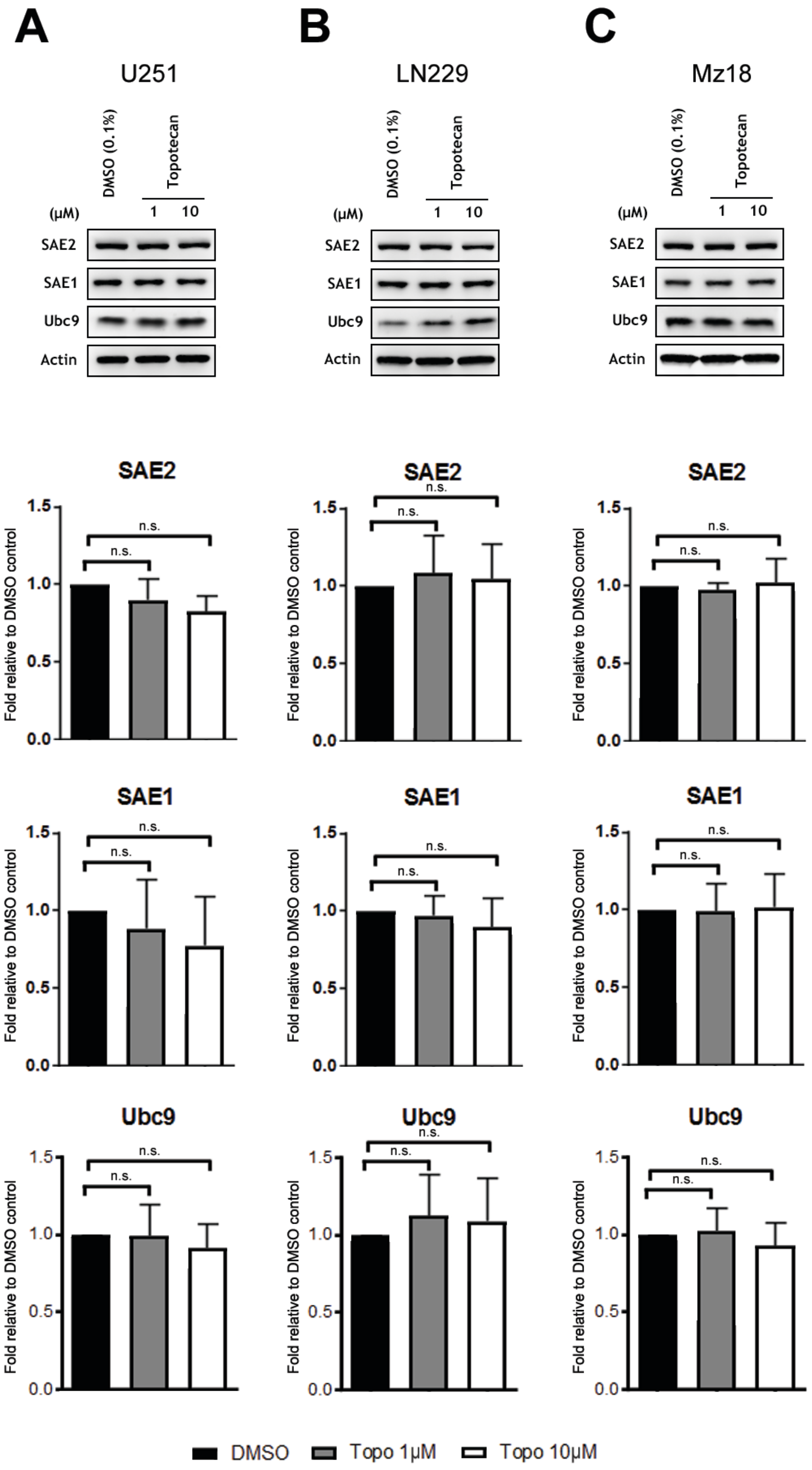

Supplemental Figure 5

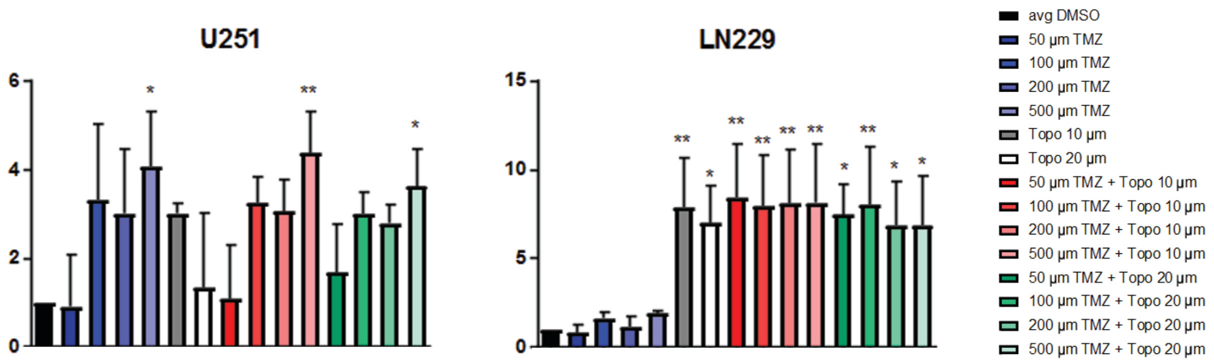

Supplemental Figure Legends:

Supp. Fig. 1. The effects of topotecan treatment on levels of selected post-translational modifications (PTMs) in GBM cell line U251

(A) NEDD8, (B) UFM1, (C) FUB1, and (D) ISG15 at concentrations of 1μM and 10μM of topotecan. Topotecan induces a significant decrease in NEDD8 conjugation at 10μM but not 1μM, and fails to induce significant changes in the conjugation of UFM1, FUB1, and ISG15. Representative immunoblots are shown. The high-molecular-weight (>100 kDa) bands corresponding to NEDD8, UFM1, and FUB1 conjugates and the bands corresponding to ISG15 conjugates (50-100 kDa) were cropped in each lane and the total intensities were measured. Densitometries were normalized to corresponding actin levels and expressed as a fold difference relative to the control (DMSO). Data are means (± SD) from n=3 independent experiments. **$p \leq 0.01$ *vs*. DMSO.

Supp. Fig. 2. The effects of topotecan treatment on levels of selected post-translational modifications (PTMs) in GBM cell line LN229

(A) NEDD8, (B) UFM1, (C) FUB1, and (D) ISG15 at concentrations of 1μM and 10μM of topotecan. Topotecan induces a significant decrease in NEDD8 conjugation at 10μM but not 1μM, and fails to induce significant changes in conjugation of UFM1, FUB1, and ISG15. Representative immunoblots are shown. The high-molecular-weight (>100 kDa) bands corresponding to NEDD8, UFM1, and FUB1 conjugates and the bands corresponding to ISG15 conjugates (50-100 kDa) were cropped in each lane and the total intensities were measured. Densitometries were normalized to corresponding actin levels and expressed as a fold difference relative to the control (DMSO). Data are means (± SD) from n=3 independent experiments. ***$p \leq 0.005$ *vs*. DMSO.

Supp. Fig. 3. The effects of topotecan treatment on levels of selected post-translational modifications (PTMs) in GBM cell line Mz18

(A) NEDD8, (B) UFM1, (C) FUB1, and (D) ISG15 at concentrations of 1μM and 10μM of topotecan. Topotecan fails to induce significant changes in the conjugation of any of these proteins. Representative immunoblots are shown. The high-molecular-weight (>100 kDa) bands

corresponding to NEDD8, UFM1, and FUB1 conjugates and the bands corresponding to ISG15 conjugates (50-100 kDa) were cropped in each lane and the total intensities were measured. Densitometries were normalized to corresponding actin levels and expressed as a fold difference relative to the control (DMSO). Data are means (± SD) from n=3 independent experiments.

Supp. Fig. 4. Topotecan fails to induce changes in the protein levels of the highly conserved E1 (SAE1/SAE2) or E2 (Ubc9) proteins

Representative immunoblots are shown. The bands corresponding to SAE1 (38 kDa), SAE2 (71.2 kDa), and Ubc9 (18 kDa) were cropped in each lane and the total intensities were measured. Densitometries were normalized to corresponding actin levels and expressed as a fold difference relative to the control (DMSO). Data are means (± SD) from n= 3 independent experiments.

Supp. Fig. 5. Cytotoxicity of temozolomide, topotecan, and temozolomide + topotecan in U251 and LN229 GBM cell lines

Cytotoxicity was measured by release of lactate dehydrogenase (LDH) into cell culture media at 72 hrs. U251 is typically considered a "TMZ-sensitive" line, whereas LN229 is typically considered a "TMZ-resistant" line. At high doses, TMZ alone and TMZ + topotecan cause significant release of LDH in U251. TMZ alone fails to cause significant LDH release in LN229, while a combination of TMZ + topotecan results in highly significant release of LDH. LDH release is reported as a fold increase relative to the control (DMSO). Data are means (± SD) from n= 3 independent experiments., *$p \leq 0.05$, **$p \leq 0.01$ vs. DMSO.
